# A novel muscle network approach for objective assessment and profiling of bulbar involvement in ALS

**DOI:** 10.3389/fnins.2024.1491997

**Published:** 2025-01-10

**Authors:** Panying Rong, Lindsey Heidrick, Gary Pattee

**Affiliations:** ^1^Department of Speech-Language-Hearing: Sciences and Disorders, University of Kansas, Lawrence, KS, United States; ^2^Department of Hearing and Speech, University of Kansas Medical Center, Kansas City, KS, United States; ^3^Neurology Associate P.C., Lincoln, NE, United States

**Keywords:** graph neural networks, masticatory muscles, speech disorders, neurodegenerative diseases, amyotrophic lateral sclerosis, surface electromyography, quantitative evaluation, biomarker

## Abstract

**Introduction:**

As a hallmark feature of amyotrophic lateral sclerosis (ALS), bulbar involvement significantly impacts psychosocial, emotional, and physical health. A validated objective marker is however lacking to characterize and phenotype bulbar involvement, positing a major barrier to early detection, progress monitoring, and tailored care. This study aimed to bridge this gap by constructing a multiplex functional mandibular muscle network to provide a novel objective measurement tool of bulbar involvement.

**Methods:**

A noninvasive electrophysiological technique—surface electromyography—was combined with graph network analysis to extract 48 features measuring the regulatory mechanisms, connectivity, integration, segregation, assortativity, and lateralization of the functional muscle network during a speech task. These features were clustered into 10 interpretable latent factors. To evaluate the utility of the muscle network as a bulbar measurement tool, a heterogenous ALS cohort, consisting of eight individuals with overt clinical bulbar symptoms and seven without, along with 10 neurologically healthy controls, was employed to train and validate statistical and machine learning algorithms to assess the disease effects on the network features and the relation of the network performance to the current clinical diagnostic standard and behavioral patterns of bulbar involvement.

**Results:**

Significant disease effects were found on most network features. The most robust effects were manifested by reduced and more variable myoelectric activities, and reduced functional connectivity and integration of the muscle network. The 10 latent factors (1) demonstrated acceptably high efficacy for detecting bulbar neuromuscular changes across all clinically confirmed symptomatic cases and clinically silent prodromal cases (area under the curve = 0.89–0.91; F1 score = 0.85–0.87; precision = 0.84–0.86; recall = 0.87–0.88); and (2) selectively correlated with clinically meaningful behavioral patterns (conditional *R*^2^ = 0.45–0.81).

**Conclusion:**

The functional muscle network shows promise for an objective quantifiable measurement tool to improve early detection and profiling of bulbar involvement across the prodromal and symptomatic stages. This tool has various strengths, including the use of a clinically readily available noninvasive instrument, fully automated data processing and analytics, and generation of interpretable objective outcome measures (i.e., latent factors), together rendering it highly scalable in routine clinical practice for assessing and monitoring of bulbar involvement.

## 1 Introduction

Neurodegenerative diseases are progressive, currently incurable conditions affecting millions of people worldwide, with an increasing prevalence owing in part to the extensions in lifespan (Gitler et al., 2017). As multisystem disorders, neurogenerative diseases progressively affect multiple domains of function, including communication (speech and language), cognition (memory and executive function), swallowing, mobility, amongst others. The clinical manifestations and progression of neurodegenerative diseases are highly heterogenous across individuals, and the relation of such clinical heterogeneity to the underlying pathophysiology remains poorly understood. This knowledge gap posits a major barrier to the development of effective therapeutic treatments. Currently, the mainstay of the management of neurodegenerative diseases focuses on controlling symptoms, slowing functional declines, preserving quality of life, and prolonging survival, through both pharmacological and nonpharmacological interventions (Church, 2021; Norris et al., 2020). To optimize the outcomes of these interventions, a patient-centered approach has been advocated, emphasizing delivering the right intervention to the right person at the right time (Miller et al., 2018; Olszewska and Lang, 2023). This approach, also known as precision or personalized medicine in a broader context (Pokorska-Bocci et al., 2014), requires a better understanding of the pathophysiological processes and phenotypes that underlie the clinical heterogeneity to improve intervention tailoring based on individual characteristics.

To better understand the pathophysiological mechanisms of neurodegenerative diseases, a network-based perspective has been posited to link three aspects of neurodegeneration: (1) the variable spreading patterns of pathogenic processes, (2) the degeneration of distributed neural networks, and (3) their integrative influence on clinical manifestations (Vogel et al., 2023). The major contribution of this perspective is that it identifies an intermediate phenotype (i.e., neural networks) to account for the clinical heterogeneity of neurodegenerative diseases. Moreover, it also paves the way for investigating the highly heterogeneous clinical manifestations and progression of neurodegenerative diseases using a well-developed, powerful mathematical tool—network analysis (Newman, 2003). This analysis maps distributed networks as nodes (e.g., neural structures) and edges (e.g., anatomical or functional connections between neural structures), and characterize nodal and edgewise properties of the network using graph-based descriptors (Rubinov and Sporns, 2010). Such descriptors provide a window into the rules governing the behaviors (e.g., regulatory mechanisms of nodal activities) and structural organization (e.g., connectivity between nodes) of the network. By establishing individualized and dynamic profiles of nodal and edgewise features, this network approach may offer an effective means to characterize and quantify the neurodegenerative processes at an individual level to improve personalized diagnosis and management.

So far network analysis has been primarily applied to study the degeneration of brain networks related to dementia syndromes, such as in Alzheimer's disease (Oxtoby et al., 2017; Vogel et al., 2023). The other functional domains remain relatively underexplored. Notably, bulbar motor function (i.e., speech and swallowing), which is vital to daily functioning, psychosocial, emotional, and physical health, has been rarely studied from the network perspective. Speech and swallowing disorders constitute key components of neurodegeneration in various conditions such as amyotrophic lateral sclerosis (ALS). Compared with other functional domains (e.g., mobility), there is a paucity of validated objective quantifiable markers of bulbar involvement (Pattee et al., 2019; Yunusova et al., 2019). This gap significantly hampers the early detection, progress monitoring, and tailored care of speech and swallowing disorders in neurodegenerative diseases and further undermines the efforts for therapeutic treatment discovery due to the lack of an efficacious means to evaluate treatment effects on bulbar motor function in clinical trials. Therefore, the need for objective quantifiable bulbar markers is well recognized (Chiaramonte et al., 2019; Green et al., 2018; Tondo and De Marchi, 2022). To fill this need, network analysis presents a huge potential in providing an efficient computational tool to quantify the performance of the bulbar motor system.

The purpose of this study is to exploit the potential of network analysis for objective bulbar marker development, by constructing a multiplex functional mandibular muscle network during speech to characterize bulbar involvement in ALS across the prodromal and symptomatic stages. Bulbar involvement is a hallmark feature of ALS, affecting the majority of patients during the course of ALS progression regardless of disease onset (e.g., bulbar/spinal) (Green et al., 2013; Yorkston et al., 1993). According to a patient-based subjective experience report, loss of useful speech secondary to bulbar involvement has been identified as the most devastating consequence of ALS, significantly impacting social participation, emotional health, and quality of life (Hecht et al., 2002). Although the current clinical standards for bulbar assessment focus on symptoms and functional outcomes, it is well established in the research literature that subclinical bulbar involvement starts long before the onset of clinical symptoms and functional declines in ALS (Rong et al., 2015a, 2016). Developing objective markers to capture such subclinical bulbar involvement across the prodromal and symptomatic stages can therefore provide important mechanistic insights for phenotyping the heterogeneous bulbar presentations and progression trajectories in ALS to improve personalized care.

While most existing network models are built upon brain imaging data, this study innovatively applied network analysis to electrophysiological data acquired by a clinically readily available, noninvasive instrumental technique—surface electromyography (sEMG). This methodological choice was contingent on three considerations. First, different from other neurodegenerative diseases such as Alzheimer's disease and Parkinson's disease, which primarily involve brain structures and pathways, ALS attacks various types of neurons in the cerebrum, brainstem, and spinal cord, as well as in the pathways connecting these central nervous system (CNS) structures with each other and with the muscles in the peripheral nervous system (PNS). A brain network model can only capture pathological changes in the CNS, but not in the PNS or in the interface between CNS and PNS. In contrast, the performance of a functional muscle network reflects the integrative functioning of all upstream CNS and PNS structures and pathways. Such a muscle network model can thus provide a more complete picture of the neuropathological mechanisms of ALS compared to a brain network model. Second, with immediate anatomical attachment to the effectors (e.g., speech articulators), the performance of a functional muscle network is directly associated with the behaviors of these effectors, which constitute an important part of oral mechanism exam in standard clinical bulbar assessment (Duffy, 2013; Yunusova et al., 2019). In this sense, the muscle network can offer explanatory insights into the complex, poorly understood clinical-neuropathological relationship in ALS, by providing an interface to link the clinically relevant behavioral patterns with the neuropathological underpinnings. Third, from a practical standpoint, the instrumental technique for constructing a functional muscle network (i.e., sEMG) is more accessible and less expensive compared with the neuroimaging techniques required for constructing a brain network. Lastly, the selected mandibular muscle groups for network construction have been demonstrated by previous studies to show measurable changes as early as during the prodromal stage of bulbar involvement in ALS (Rong and Jawdat, 2021; Rong and Pattee, 2021, 2022; Rong and Rasmussen, 2024). Taken together, these practical and empirical considerations support the potential of a sEMG-based functional mandibular muscle network for a clinically scalable objective bulbar assessment tool.

To construct the proposed multiplex functional mandibular muscle network, we developed a fully automated data processing and analytic approach to characterize the network performance using graph-based measures. We hypothesized that these measures would (1) effectively detect and profile bulbar involvement across the prodromal and symptomatic stages in ALS and (2) associate with clinically relevant behavioral patterns of the effector (i.e., jaw).

## 2 Materials and methods

This study was part of an ongoing larger-scope project. The protocol of this project was approved by the Institutional Review Board of the university medical center. Written informed consent was obtained from all participants. All study procedures were non-invasive and minimal risk. No adverse events were reported.

### 2.1 Participants

Fifteen individuals with ALS (nine men/six women; age: 38–77 years), including eight with overt clinical bulbar symptoms and seven without, and 10 neurologically healthy controls (three men/seven women; age: 38–81 years) participated in this study. The inclusionary criteria included: (1) being diagnosed with definite or probable ALS as per the revised El Escorial Criteria (Brooks et al., 2000) for participants with ALS or reporting no known neurological diseases or injury for healthy controls; (2) speaking American English as the first and primary language; (3) passing hearing screening at 1,000, 2,000, and 4,000 Hz at 30 dB in the better ear; (4) possessing adequate cognitive function to follow instructions and perform experimental tasks as per by standard cognitive screening procedures.

Participants with ALS were recruited from the multidisciplinary ALS clinic of the university medical center. As per standard clinical examination procedures (e.g., oral mechanism exam, clinician-based screening/evaluation, patient-reported outcomes), eight of the participants presented with overt clinical bulbar symptoms, providing samples representative of the symptomatic stage of bulbar involvement. The other seven participants exhibited no overt clinical bulbar symptoms. According to a prior longitudinal study (Rong et al., 2015a), the likelihood that these participants, who were on average 453 days post-diagnosis, had experienced subclinical bulbar involvement at the time of enrolling into this study was high despite the absence of clinical symptoms. Thus, these participants were regarded as samples representing the prodromal stage of bulbar involvement. Together, the inclusion of both prodromal and symptomatic cases offered a comprehensive sample set representative of the whole spectrum of bulbar involvement in ALS to address the research aims.

To evaluate the severity of the overall and bulbar functional disabilities, all participants with ALS completed the ALS Functional Rating Scale-Revised (ALSFRS-R) (Cedarbaum et al., 1999). ALSFRS-R is the most widely accepted means of monitoring disabilities in ALS clinical practice and clinical trials. It consists of 12 questions that assess four domains of function, including gross motor, fine motor, bulbar, and respiratory functions, with each question being rated on a scale of 0 to 4 points (4 being normal; 0 being the highest level of disability). Based on the participant's responses, the total score for all 12 questions and the bulbar subscore for the three questions related to bulbar items (i.e., speech, salivation, swallowing) were calculated to index the overall and bulbar functional disabilities, respectively. The clinical, functional, and demographic characteristics of the participants are provided in Supplementary Table S1, and a statistical summary of these characteristics is displayed in Table 1.

**Table 1:** Summary of demographic, clinical, and functional characteristics of participants.

**Participant characteristics**	**ALS (*N* = 15)**	**Control (*N* = 10)**	**Statistics for between-group comparison**
**Demographic**			
Women (*n* %)	40.00%	70.00%	χ^2^ = 1.13, *p* = 0.29
Age, years (M; SD)	60.73; 12.66	66.80; 13.02	*F*(1, 23) = 0.54, *p* = 0.47
**Clinical**			
Disease onset (*n* %)	Bulbar: 33.33% Spinal: 66.67%	N/A	N/A
Stage of bulbar involvement (*n* %)	Prodromal: 46.67% Symptomatic: 53.33%	N/A	N/A
Days since diagnosis (M; SD)	382.93; 416.72	N/A	N/A
**Functional**			
ALSFRS-R: total score (M; SD)	37.33; 6.55	N/A	N/A
ALSFRS-R: bulbar sub-score (M; SD)	9.60; 2.87	N/A	N/A

ALS, amyotrophic lateral sclerosis; control, healthy controls; ALSFRS-R, ALS Functional Rating Scale-Revised.

### 2.2 Experimental task, setup, and data collection

To construct the proposed functional muscle network, the myoelectric activities of three bilateral pairs of jaw muscles—anterior temporalis (TEMP), masseter (MAS), anterior belly of digastric (ABD)—were recorded by a wireless sEMG system (BIOPAC) during a speech task. The selection of target muscles was primarily based on accessibility consideration. The morphological characteristics of anterior temporalis, masseter, and anterior belly of digastric muscles make them easily accessible and reliably measurable by surface electrodes, as demonstrated by a body of experimental work by our research team and others (Koole et al., 1991; Moore et al., 1988; Rong and Pattee, 2021).

Regarding task selection, speech production, as a fine oromotor behavior, requires rapid contractions and complex coordination of a variety of bulbar muscles. Speech tasks such as oral reading and narrative have been consistently demonstrated by prior studies to be sensitive for detecting subtle bulbar involvement (e.g., during the prodromal stage) in ALS (Rong and Heidrick, 2022, 2024; Rong and Pattee, 2022). In this study, we selected an oral reading task, where participants read a standard phonetically balanced reading passage, namely Rainbow Passage, at their habitual rate and loudness. This passage consists of 19 sentences with a total of 329 words, which cover the entire phonetic inventory with the frequency of each phoneme matching their distribution in English. Oral reading of the Rainbow Passage has proven by prior work to elicit robust activations of mandibular muscles in both neurologically healthy and impaired speakers (Rong and Jawdat, 2021; Rong and Pattee, 2022). The theoretical and empirical evidence base provides the rationale for our task selection to enhance both the robustness and generalizability of the results of this study.

To maximize reproducibility and minimize artifacts (e.g., crosstalk), we followed the best practice guidelines for surface electrode selection, setup, and recording (Castroflorio et al., 2008; Merletti and Muceli, 2019; Stepp Cara, 2012). First, the target areas of skin were prepared using an alcohol swab to increase skin conductance. Following skin preparation, self-adhesive bipolar Ag/AgCl electrodes with 11 mm diameter size and the closest inter-electrode distance possible (e.g., about 20 mm) were attached to the skin over the belly of each target muscle, parallel to fiber orientation. To enhance the reproducibility of this procedure, craniofacial anatomical landmarks were used to guide the placement of electrodes along the cantho-gonial line for masseter, vertically at the coronal suture for anterior temporalis, and submentally along the posterior-inferior direction for anterior belly of digastric. Ground electrodes were attached to the participant's shoulder. Electrode placement was verified by calibration gestures such as jaw oscillation and clenching to ensure data validity. The analog signals acquired by the surface electrodes were pre-amplified by 2,000 and band-passed filtered at 5–500 Hz by the wearable BioNormadix modules, digitized at 2,000 Hz by the MP160 module, and finally recorded by the Acknowledge software.

To examine the relation of the proposed functional mandibular muscle network to behavioral patterns of the jaw, jaw motion was recorded in three dimensions by an electromagnetic tracking system (Wave, Northern Digital Inc.). During the recording, participants were seated next to a field generator to the right; a small wired sensor was attached to the center of lower chin, and a reference sensor was attached to the center of forehead for head movement correction. Jaw motion was captured by the sensor on the lower chin relative to the forehead and was recorded at 100 Hz by the WaveFront software. Additionally, the midsagittal contour of the hard palate was traced by a manufacturer-supplied probe, from the posterior edge at the intersection between the hard and soft palate to the anterior edge at the location of the lower central incisor. This palatal trace served as an anatomical reference to characterize the motion pattern of the jaw in the subsequent kinematic analysis.

**Figure 1:**
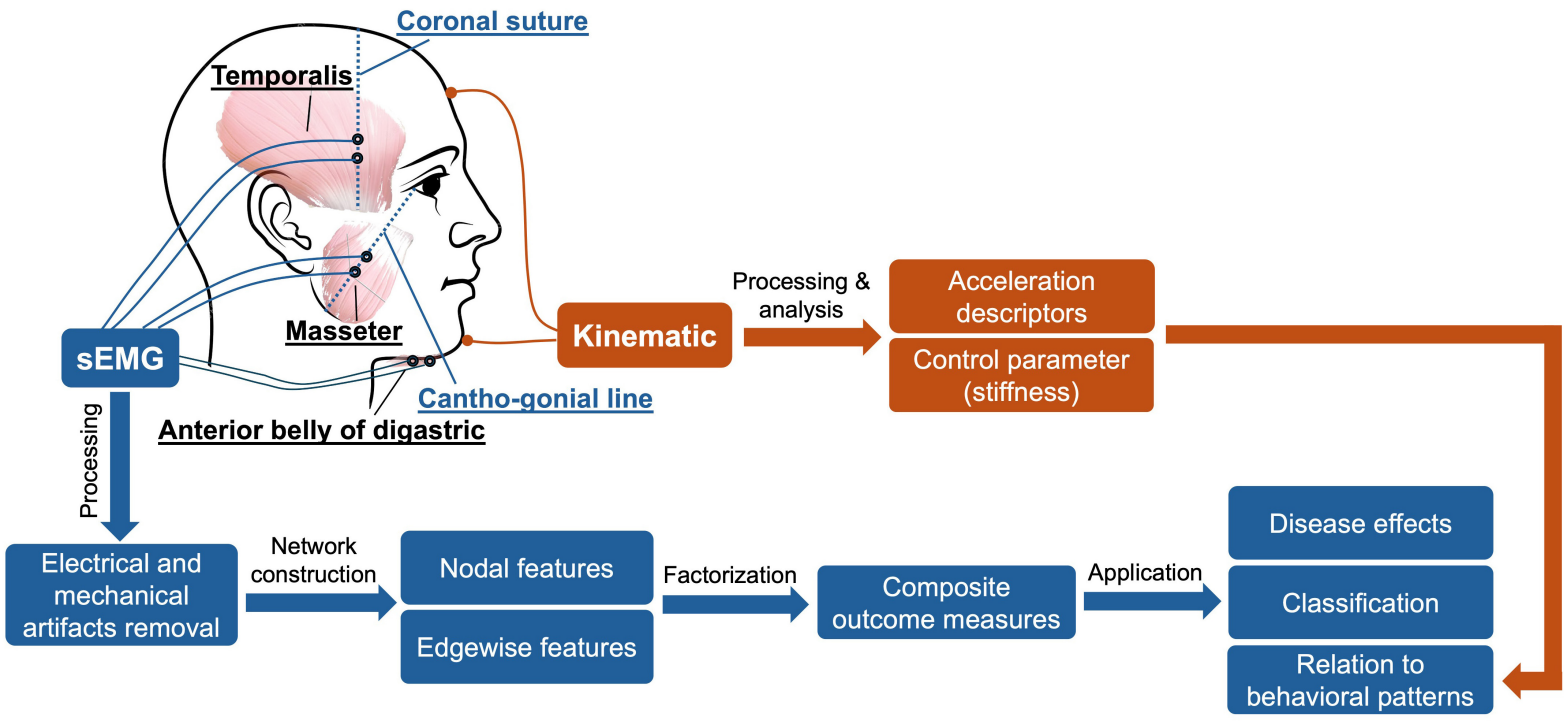
Overview of experimental paradigm.

In addition to the sEMG and kinematic recordings, speech was audio-recorded by a head-mounted microphone (DPA dfine 4188) placed ~5 cm away from the left lip corner. The audio signal was preprocessed by the Behringer Xenyx 802 sound conditioner and recorded at 22,050 Hz by the WaveFront software, simultaneously with the kinematic signal. The audio recording served as a reference to assist with sEMG and kinematic data segmentation and interpretation. Figure 1 provides an overview of the experimental paradigm along with the procedures for signal processing and analysis, as elaborated below.

### 2.3 sEMG data processing

sEMG is known to be susceptible to various sources of electrical and mechanical artifacts (e.g., motion at the skin-electrode interface, crosstalk, power line noise). To enhance the signal-to-noise ratio of the sEMG data, we used a multistep signal processing approach to minimize these artifacts, following the recommended guidelines in the sEMG literature (De Luca et al., 2010; Rong and Pattee, 2022; Stepp Cara, 2012). First, to minimize the effect of crosstalk, we adopted a blind source separation (BSS) algorithm from Kilner et al. (2002) to remove potential contamination of nearby muscles from each sEMG channel. BSS is a statistical signal processing technique to reconstruct the original unobserved signals (e.g., sources reflecting true myoelectric activities) based on the decomposition of the measured signals (i.e., surface-detected signals) (Talib et al., 2019). BSS has been successfully applied to reduce crosstalk from facial sEMG recordings in previous research (Sato and Kochiyama, 2023) and is therefore adopted by this study to serve the same purpose. After crosstalk removal, all sEMG channels were notch-filtered at 60 Hz and high-pass filtered at 20 Hz to further remove power line noise and low-frequency movement artifacts, respectively. Lastly, DC offsets were removed from each channel.

### 2.4 sEMG data analytics

**Figure 2:**
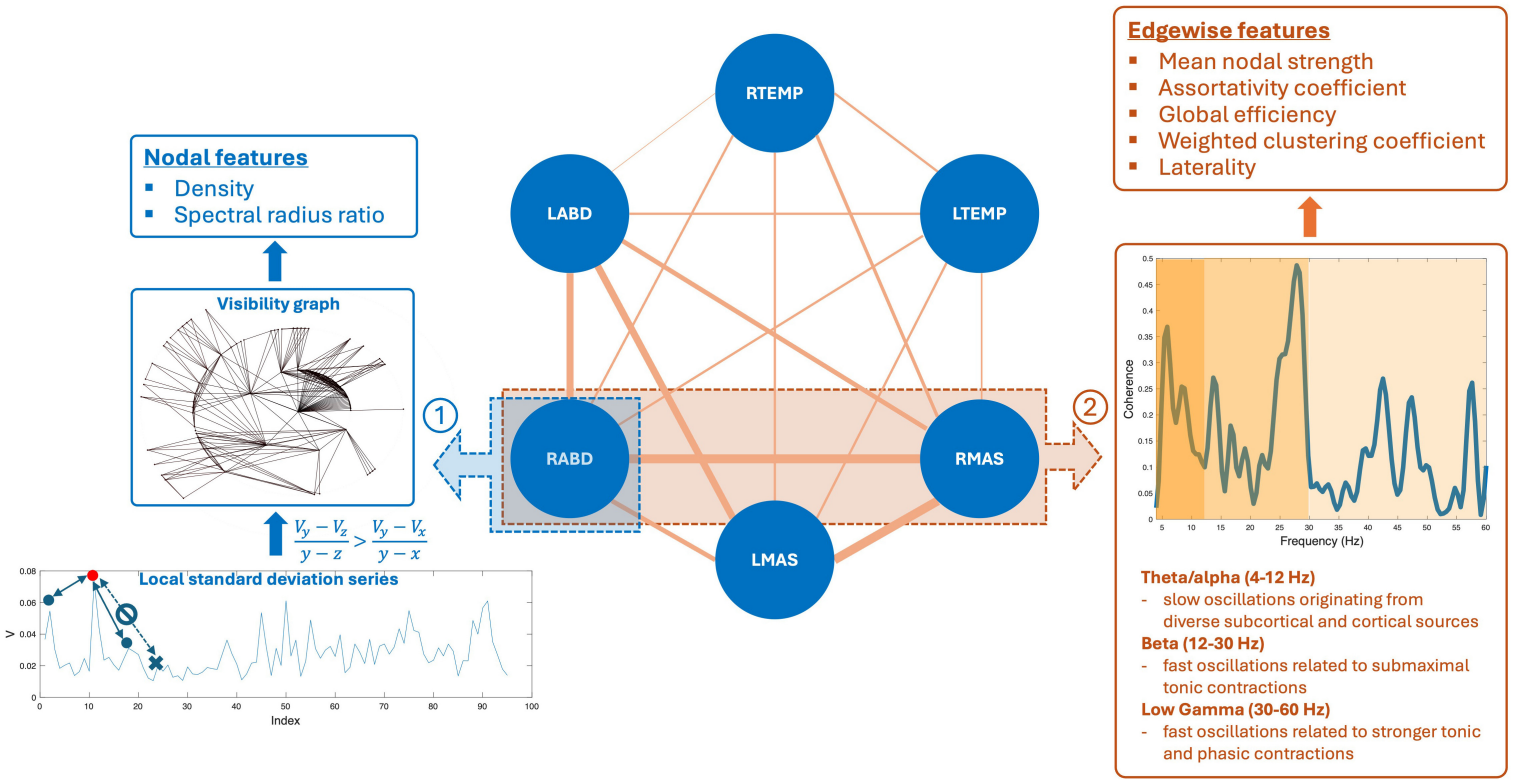
Pipeline for constructing the multiplex functional mandibular muscle network. The network consists of six nodes corresponding to six mandibular muscles: right temporalis (RTEMP), left temporalis (LTEMP), right masseter (RMAS), left masseter (LMAS), right anterior belly of digastric (RABD), left anterior belly of digastric (LABD). Each pair of nodes are connected by an edge, with the weight of the edge (denoted by line width) reflecting the strength of functional connection between muscles (measured by intermuscular coherence) in three frequency bands (theta/alpha, beta, low gamma). Step ①: Nodal feature extraction. Using the RABD node as an example for demonstration, the surface electromyography signal for the node is first converted to a local standard deviation series, which is then transformed to a visibility graph. Based on this graph, two visibility descriptors—density and spectral radius ratio—are derived to measure the overall level and variability of nodal activity, respectively. Step ②: Edgewise feature extraction. Using the edge between the RABD and RMAS nodes as an example, the magnitude-squared coherence spectrum is calculated; after verifying significance, the mean coherence within the target frequency bands (highlighted by different colors) is computed. Using these coherence values as edge weights, a set of network measures, including mean nodal strength, assortativity coefficient, global efficiency, weighted clustering coefficient, and laterality, are derived to characterize the edgewise features of the muscle network.

Based on the processed sEMG signals, a weighted multiplex functional mandibular muscle network was constructed. This network consisted of six nodes, each representing a muscle; the weights of the edge between nodes reflected the functional connection between each pair of muscles in different frequency bands (see details below). To characterize the structural organization and behaviors of the network in relation to bulbar neuromuscular pathology in ALS, a fit-for-purpose data analytic program was developed and implemented in MATLAB (R2023a) to extract a variety of nodal and edgewise features from the sEMG signals for each sentence of the Rainbow Passage [total *N* = (25 participants × 19 sentences) − 2 errors = 473]. An overview of these network features is provided in Table 2, and the pipeline for feature extraction is outlined in Figure 2, with methodological details described below.

**Table 2:** Overview of network features.

**Type**	**Feature**	**Notation**	**Targeted functional underpinnings**
Nodal	density_RTEMP density_LTEMP density_RMAS density_LMAS density_RABD density_LABD	Density	Level of muscle activity
	specradRatio_RTEMP specradRatio_LTEMP specradRatio_RMAS specradRatio_LMAS specradRatio_RABD specradRatio_LABD	Spectral radius ratio	Variability of muscle activity
Edgewise	nodstr_theta.alpha nodstr_beta nodstr_gamma	Mean nodal strength	Overall functional connectivity of multiplex muscle network
	ac_theta.alpha ac_beta ac_gamma	Assortativity coefficient	Selective functional connectivity of multiplex muscle network
	ge_theta.alpha ge_beta ge_gamma	Global efficiency	Functional integration of multiplex muscle network
	wcc_RTEMP_theta.alpha wcc_LTEMP_theta.alpha wcc_RMAS_theta.alpha wcc_LMAS_theta.alpha wcc_RABD_theta.alpha wcc_LABD_theta.alpha	Weighted clustering coefficient	Functional segregation of multiplex muscle network
	wcc_RTEMP_beta wcc_LTEMP_beta wcc_RMAS_beta wcc_LMAS_beta wcc_RABD_beta wcc_LABD_beta		
	wcc_RTEMP_gamma wcc_LTEMP_gamma wcc_RMAS_gamma wcc_LMAS_gamma wcc_RABD_gamma wcc_LABD_gamma		
	lat_TEMP_MAS_theta.alpha lat_TEMP_ABD_theta.alpha lat_MAS_ABD_theta.alpha	Laterality index	Functional lateralization of multiplex muscle network
	lat_TEMP_MAS_beta lat_TEMP_ABD_beta lat_MAS_ABD_beta		
	lat_TEMP_MAS_gamma lat_TEMP_ABD_gamma lat_MAS_ABD_gamma		

#### 2.4.1 Nodal features

Nodal features were extracted from each sEMG channel to characterize the regulatory mechanisms for modulating the myoelectric activity of each jaw muscle. To this end, each sEMG signal was first transformed into a local standard deviation series using Equation 1:


(1) $$\begin{array}{l} V_{j}=\sqrt{\frac{\sum_{\left(j-1\right)\;*\;L+1}^{jL}{\left(U_{i}-{Ū}_{j}\right)}^{2}}{L-1}} \end{array}$$

where [*U*_*j*_] = [*U*_1_, *U*_2_, …, *U*_*N*_] is the original sEMG signal, [*V*_*j*_] = [*V*_1_, *V*_2_, …, *V*_*M*_] is the transformed local standard deviation series, and *L* is the length of interval for standard deviation calculation, which was set to 50 ms (i.e., *L* = 100), in line with our prior study (Rong et al., 2024). These local standard deviation series were then transformed to visibility graphs. This transformation treated each data point in the series as a node and determined the connection between nodes based on Equation 2:


(2) $$\begin{array}{l} \frac{V_{y}-V_{z}}{y-z}>\frac{V_{y}-V_{x}}{y-x} \end{array}$$

where *V*_*z*_ is an arbitrary node between two given nodes *V*_*x*_ and *V*_*y*_. If all nodes between *V*_*x*_ and *V*_*y*_ meet the criterion in Equation 2, *V*_*x*_ and *V*_*y*_ are regarded as being connected. A node corresponding to a local peak in the series (i.e., a state of high-level activation) tends to have high connectivity with its neighboring nodes (i.e., high visibility). This notion relates the structure of the visibility graph with the dynamics of myoelectric activities, allowing the descriptors of the graph to inform the regulatory mechanisms for modulating myoelectric activities.

To characterize the structure of the visibility graph, two descriptors—density and spectral radius ratio—were derived. Density represents the overall node connectivity of the graph, which is defined as the ratio of the total number of the graph's edges to the largest possible number of edges, as per Equation 3:


(3) $$\begin{array}{l} density=\frac{2\times m}{M\left(M-1\right)} \end{array}$$

where *M* and *m* are the total number of nodes and edges of the graph, respectively. Spectral radius ratio represents the variation of node connectivity, which is defined as the ratio of the principal eigenvalue of the adjacency matrix of the graph to the mean node degree (Meghanathan, 2014).

Together, density and spectral radius ratio provided quantitative means to assess the overall level and variability of myoelectric activities. Given the well documented changes in motor unit recruitment and firing patterns in ALS (de Carvalho et al., 2014, 2012), myoelectric activities tend to be reduced and become more irregular, as observed in prior research (Rong and Pattee, 2022). Density and spectral radius ratio were intended to target and quantify these changes related to the nodal properties of the muscle network.

#### 2.4.2 Edgewise features

Edgewise features were calculated based on intermuscular coherence in three frequency bands—theta/alpha (4–12 Hz), beta (12–30 Hz), and low gamma (30–60 Hz). By definition, coherence is a measure of temporal correlation between two signals in the frequency domain (Laine and Valero-Cuevas, 2017; Reyes et al., 2017). It is well known that neural oscillations in different frequency bands are related to distinct neural drives for modulating muscle functions. Specifically, beta and low-gamma oscillations originate directly from the motor cortical network, which have been associated with submaximal tonic contractions and attentionally more demanding, stronger tonic and phasic contractions, respectively (Boonstra et al., 2015; Brown, 2000; Laine et al., 2015). Theta/alpha oscillations have been linked to diverse subcortical and cortical sources (e.g., brainstem, sensory cortex) outside of the motor cortical network, contributing to indirect motor control (e.g., sensorimotor integration and adaptation) (MacKay, 1997; Maezawa, 2017; Suppa et al., 2016). Therefore, measurements of intermuscular coherence in these bands can provide a window into the multiplex functional connection between muscles related to different neural sources. The feasibility of measuring intermuscular coherence in these bands from sEMG signals has been established in the literature in both neurologically healthy and impaired individuals (Carlsen et al., 2023; Fisher et al., 2012; Flood et al., 2019; Issa et al., 2017; Laine and Valero-Cuevas, 2017; Rong and Pattee, 2021).

To calculate intermuscular coherence, all sEMG signals were full wave rectified to maximize the timing information about muscle activation (Halliday et al., 1995). The rectified signals were then reconstructed by identifying and concatenating stationary 1-s epochs around the bursts for each channel. Magnitude-squared coherence spectrum was calculated based on the reconstructed signals for each pair of muscles, using Equation 4:


(4) $$\begin{array}{l} {\left|R_{xy}\right|}^{2}=\frac{{{|S}_{xy}\left(f\right)|}^{2}}{S_{xx}{\left(f\right)S}_{yy}\left(f\right)} \end{array}$$

where ${\left|{\text{R}}_{\text{xy}}\right|}^{2}$ is magnitude-squared coherence spectrum, S_xy_(*f*) is the cross-spectrum between two muscles, and S_xx_(*f*) and S_yy_(*f*) are the auto-spectra for each muscle. This analysis was implemented by a 4,096-point Fast Fourier Transform applied over a sliding 1,024-point Hamming window with 75% overlap, following the recommendations by Terry and Griffin (2008).

For each coherence spectrum, a significance level corresponding to the upper 95% confidence limit under the hypothesis of independence between muscles was calculated as: $S=1-0.05^{1/\left(\hat{L}-1\right)}$, where $\hat{L}$ is the adjusted number of overlapped segments in coherence calculation (Halliday et al., 1995; Terry and Griffin, 2008). Weak, non-significant coherence usually implies a spurious connection between muscles; such a connection could obscure the topology of strong, significant connections in the muscle network (Rubinov and Sporns, 2010). Therefore, the significance level was applied as a threshold to rule out spurious connections. Within each target band (theta/alpha, beta, gamma), if there was a lack of peaks above the significance level, the coherence for the band was set to zero; otherwise, the mean coherence within the band was calculated. Finally, all coherence values were transformed to Fisher *z*-scores for variance stabilization (Halliday et al., 1995).

Following the procedures above, a total of 45 coherence metrics (15 edges × 3 bands) were extracted for the multiplex functional muscle network. Using these metrics as the weights of the network's edges, graph network analysis was applied to derive a set of graph descriptors to characterize the edgewise properties of the network. These descriptors included mean nodal strength, assortativity coefficient, global efficiency, and weighted clustering coefficients.

Mean nodal strength is the average sum of edge weights across nodes, which reflects the overall functional connectivity of the network. There is a mounting body of evidence showing impaired functional connection between muscles in individuals with ALS, especially in the beta band (Fisher et al., 2012; Issa et al., 2017; Rong and Pattee, 2021). Mean nodal strength was intended to detect and quantify such impairments of functional connection in ALS.

Assortativity coefficient measures the correlation of the (weighted) degrees of all nodes between two opposite sides of an edge, providing an insight into selective functional connectivity of the network. In general, an assortatively mixed network (i.e., characterized by mutually interconnected high-degree hubs) tends to have a positive coefficient, whereas a disassortative network usually has a negative coefficient. Prior studies have reported selective vulnerability of functional connection between different muscle groups in individuals with ALS (Rong and Jawdat, 2021; Rong and Pattee, 2021). Such selective vulnerability may differentially affect the connectivity between different nodes and in turn influence the assortativity of the muscle network.

Global efficiency is the average inverse shortest path length between all pairs of nodes, which provides a global index of functional integration of the network. Weighted clustering coefficient represents the connectivity of each node to its neighboring nodes, which can be interpreted as a measure of functional segregation/specialization, that is, the ability to perform specialized functions by segregated muscle groups within the network. A complex, high-performing biological system should be both functionally integrated and specialized (Tononi et al., 1998). This notion, applied to the functional muscle network in this study, implies that the activity of functionally segregated muscle groups (e.g., agonists vs. antagonists) should be integrated, in order to generate complex, coherent, and adaptive behaviors during speech. Yet, prior work has reported overall less complex, less coherent, and more irregular speech behaviors in individuals with ALS (Rong, 2021). These changes could be attributed to impaired functional integration and/or segregation of the speech motor system due to neurodegeneration. Along this line, global efficiency and weighted clustering coefficient were employed to provide complementary insights into functional integration and segregation of the muscle network.

Besides the standard graph descriptors above, another set of edgewise features was extracted to characterize the laterality of the multiplex functional muscle network. Here laterality was indexed by the difference between the weight of a specified edge on the right side and its counterpart on the left side, normalized by the sum of all edge weights in the network. This index was calculated for three types of edges, which connected temporalis with masseter, temporalis with digastric, and masseter with digastric, respectively. These indices evaluated the functional lateralization of one agonist (temporalis-masseter) and two antagonist (temporalis-digastric, masseter-digastric) muscle groups within each target band. While the majority of bulbar muscles are bilaterally innervated, a contralateral dominance has been reported in beta-band corticomuscular coherence in previous studies, suggesting that a larger proportion of corticobulbar projection comes from the contralateral hemisphere (Maezawa, 2017; Maezawa et al., 2014). For individuals with ALS, a recent evolutionary perspective of neurodegeneration has associated evolutionary hemisphere specialization with preferential involvement (Henderson et al., 2019). It posits that ALS tends to preferentially involve cerebral structures and pathways that have evolved recently in human evolution, including those in the dominant left hemisphere serving later evolved functions such as speech (Henderson et al., 2019). Together, the interplay between the disease-related asymmetrical cerebral involvement and contralateral dominance of corticobulbar projection may result in a decrease in right-to-left functional lateralization of the muscles. The laterality index was intended to capture such a change in functional lateralization.

### 2.5 Kinematic data processing and analysis

The 3D jaw sensor data were low-pass filtered at 15 Hz by a second-order, zero-lag Butterworth filter to remove high-frequency movement artifacts. The Euclidian distance between the jaw sensor and the lower central incisor (i.e., anterior edge of the palatal trace) was calculated at each sampled time point, generating a data series representing the global movement pattern of the jaw.

This data series was submitted to a custom-developed analysis to extract three metrics: acceleration time, mean acceleration, and stiffness. Acceleration time and mean acceleration are temporal and spatial descriptors of the acceleration profile. Scientifically, according to Newton's second law, force is the product of mass and acceleration. As such, descriptors of the jaw acceleration profile can provide insights into the force exerted by the jaw muscles on the mandible. Clinically, the slowness of orofacial movement is one of the most prevalent signs of bulbar involvement in ALS (Green et al., 2013; Yorkston et al., 1993); such a sign can manifest in the acceleration profile as increased acceleration time and reduced mean acceleration, as demonstrated by prior research (Bandini et al., 2018; Rong and Heidrick, 2021). Linking these acceleration descriptors with the network measures can inform the relation of the proposed functional muscle network to the force generation capacity of the mandibular system.

From the perspective of dynamical systems, the generation of the desired force/acceleration for a specific task requires the neuromuscular system to modulate the dynamic properties of the effectors based on task demands. Along this line, several theoretical models have been established to characterize dynamic speech behaviors with a spring-mass second-order dynamical system. These models, including the original task dynamic model proposed by Saltzman and Munhall (1989) and various extended versions (Parrell and Lammert, 2019; Simko and Cummins, 2010), posit that force/acceleration is associated with two dynamic parameters—stiffness and damping. Given that these two parameters are not mutually exclusive (i.e., damping is associated with stiffness and mass), we focused on stiffness in this study. The functional significance of stiffness is well established in the motor speech literature. Increased stiffness is associated with various mechanical advantages for generating rapid movement and/or maintaining stability against perturbation to ensure movement precision (Humphrey and Reed, 1983; Moore, 1993; Moore et al., 1988). As such, linking stiffness with the network measures can inform the relation of the functional muscle network to the dynamic control mechanism of the mandibular system.

To obtain these kinematic/dynamic metrics, the first and second derivatives of the jaw Euclidian distance data series were calculated, resulting in two new data series representing velocity and acceleration traces, respectively. Acceleration time was calculated as the average duration from the onset (i.e., velocity = 0) to the peak velocity of movement across all jaw opening and closing cycles during the speech stimulus. Mean acceleration was calculated as the average acceleration across the accelerating phase of all jaw opening and closing cycles. Unlike the acceleration descriptors, stiffness was not directly measurable from the kinematic recording but can be estimated by empirical means. In this study, stiffness was estimated by the ratio of maximum speed to maximum distance of motion (Berry, 2011).

### 2.6 Statistical and computational analyses

All analyses were conducted in the R statistical computing program (R Core Team, 2022). For statistical analysis, the significance level was set to *p* < 0.05 for main effects and was adjusted using the Bonferroni method for *post-hoc* tests.

#### 2.6.1 Data reduction

As shown in Table 2, a total of 48 network features were extracted, which combined characterized the structural organization and behaviors of the multiplex functional muscle network. To reduce the dimensionality of the feature set, a data reduction technique was applied. This procedure aimed to prevent overfitting of the subsequent machine learning (ML) analyses due to the dilemma between a large number of variables and a small set of training samples, as commonly encountered in health data applications (Berisha et al., 2021). To this end, maximum likelihood factor analysis (*factanal*) with *oblimin* rotation was applied to the feature set to cluster the features that shared common variance into a lower-dimensional set of latent factors, where the number of factors was determined by parallel analysis (*fa.parallel*; Revelle, 2020). The fit of the factorization model was verified by chi-square goodness of fit test. After the verification, the scores of all factors were calculated using the *tenBerge* method. These scores represented the neuromuscular performance of the participants in the new multidimensional factor space.

#### 2.6.2 Utility of the multiplex functional muscle network for detecting and profiling bulbar involvement in ALS

To determine the utility of the multiplex functional muscle network for detecting and profiling bulbar involvement in ALS, we first evaluated the disease effect on all network features using linear mixed effects (LME) models. For nodal features, the LME models were constructed with group (i.e., ALS vs. healthy control), node (i.e., six muscles), and group-by-node interaction as the fixed effects and a subject-dependent intercept as the random effect. *Post-hoc* between-group comparisons were conducted by node based on estimated marginal means (*emmeans*; Lenth, 2020). For edgewise features, the LME models were constructed with group, band (i.e., theta/alpha, beta, gamma), and group-by-band interaction as the fixed effects and a subject-dependent intercept as the random effect. *Post-hoc* between-group comparisons were conducted by band based on estimated marginal means.

In the second step, we further evaluated the efficacy of the factor scores as derived above for classification between the ALS and healthy control samples, using supervised ML algorithms. Three ML algorithms were employed, including random forest (RF), support vector machine (SVM) with the radial basis function kernel, and k-nearest neighbors (KNN), to allow for comparison of classification performance. RF is an ensemble ML algorithm that uses a combination of decision trees to solve classification or regression problems (Breiman, 2001). SVM is a flexible ML algorithm that allows raw data to be mapped into linear or nonlinear space using different kernels (Drucker et al., 1997). KNN relies on the similarity of data points (i.e., “nearest neighbors”) to assign labels (for classification) or values (for regression) (Taunk et al., 2019). All classification models were cross-validated through five-fold cross-validation repeated 10 times, and model performance was evaluated by precision (i.e., ratio of true positives to total predicted positives), recall (i.e., ratio of true positives to total actual positives), and F1 score (i.e., harmonic mean of precision and recall, defined as $F1=2\times \frac{Precision\times Recall}{Precision+Recall}$). Moreover, the Receiver Operating Characteristic (ROC) curve and the area under the curve (AUC) were calculated for each model.

#### 2.6.3 Relation of the multiplex functional muscle network to clinically relevant behavioral patterns of the jaw

A stepwise approach was chosen to construct LME models for estimating the relation of the multiplex functional muscle network to the three kinematic/dynamic metrics of the jaw. For each metric, an initial LME model was constructed with group and the scores of all latent factors as the fixed effects, and a subject-dependent intercept as the random effect. Next, the fixed effects of the initial model were screened in a backward stepwise fashion to optimize the Akaike Information Criterion. The marginal and conditional *R*^2^ of the final models were calculated to evaluate model fit.

## 3 Results

### 3.1 Factorization

**Figure 3:**
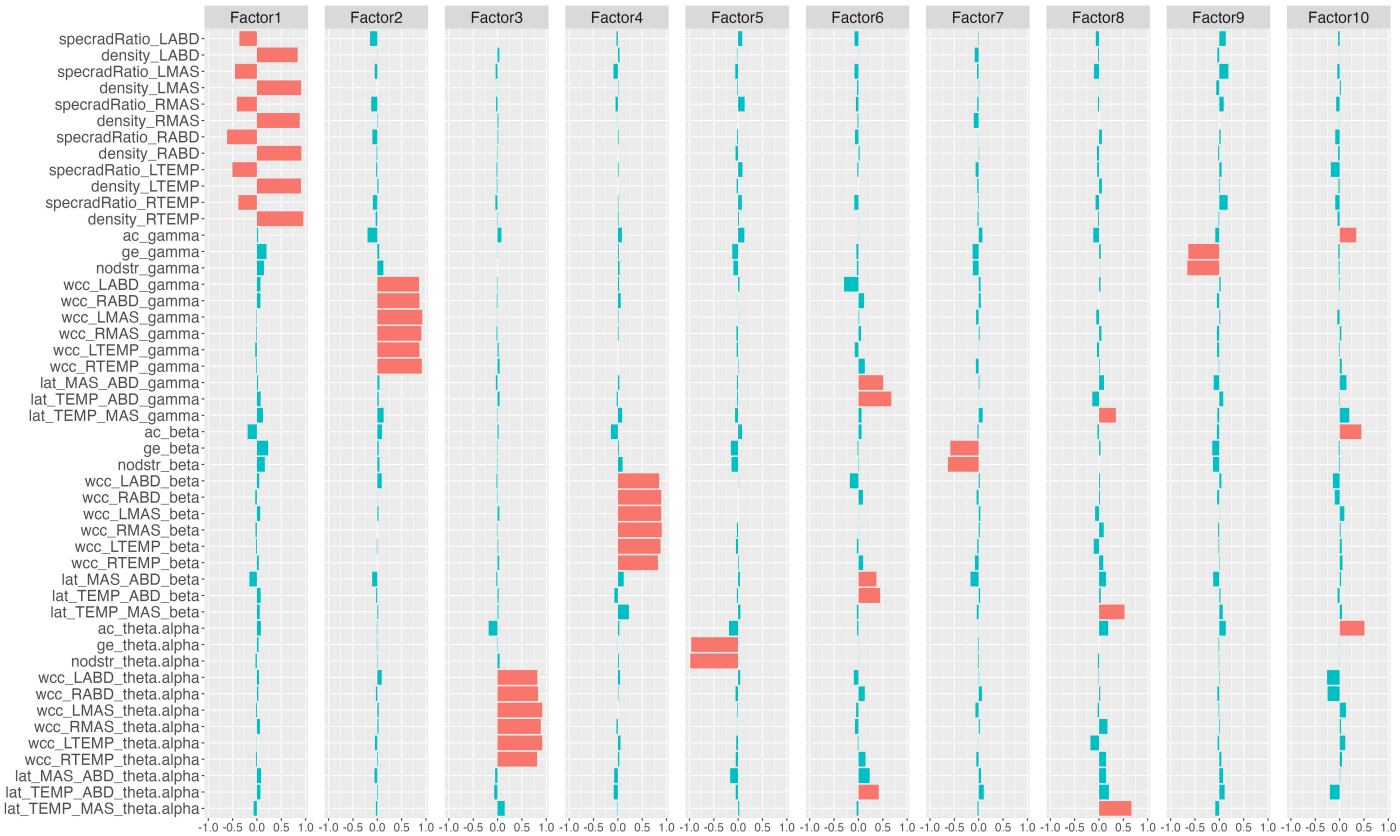
Rotated factor loadings. The height of the colored bars reflects the loading of the network features on each factor. Features that load >0.3 (i.e., absolute loading >0.3) on each factor are by convention regarded as the primary component features of the factor and are marked in red; the remaining features are marked in blue. specradRatio, spectral radius ratio; nodstr, mean nodal strength; ac, assortativity coefficient; ge, global efficiency; wcc, weighted clustering coefficient; lat, laterality index. RTEMP, right temporalis; LTEMP, left temporalis; RMAS, right masseter; LMAS, left masseter; RABD, right anterior belly of digastric; LABD, left anterior belly of digastric.

The factor analysis clustered the 48 network features into 10 latent factors. The rotated factor loadings are shown in Figure 3. This 10-factor model was determined to be sufficient based on chi-square goodness of fit test, χ^2^(693) = 4, 714.69, *p* = 0. The cumulative variance accounted for by the model was 68.2%.

Based on the rotated factor loadings, features that loaded >0.3 on each factor were conventionally identified as the primary component features of the factor. As such, the primary component features of the 10 factors were identified as: all nodal features for factor 1; gamma-band weighted clustering coefficients for factor 2; theta/alpha-band weighted clustering coefficients for factor 3; beta-band weighted clustering coefficients for factor 4; theta/alpha-band mean nodal strength and theta/alpha-band global efficiency for factor 5; laterality indices for the antagonist muscle groups (i.e., temporalis-digastric; masseter-digastric) across all three bands for factor 6; beta-band mean nodal strength and beta-band global efficiency for factor 7; laterality indices for the agonist muscle group (i.e., temporalis-masseter) across all three bands for factor 8; gamma-band mean nodal strength and gamma-band global efficiency for factor 9; assortativity coefficients across all three bands for factor 10.

### 3.2 Utility of the multiplex functional muscle network for detecting and profiling bulbar involvement in ALS

#### 3.2.1 Disease effects on network features

**Figure 4:**
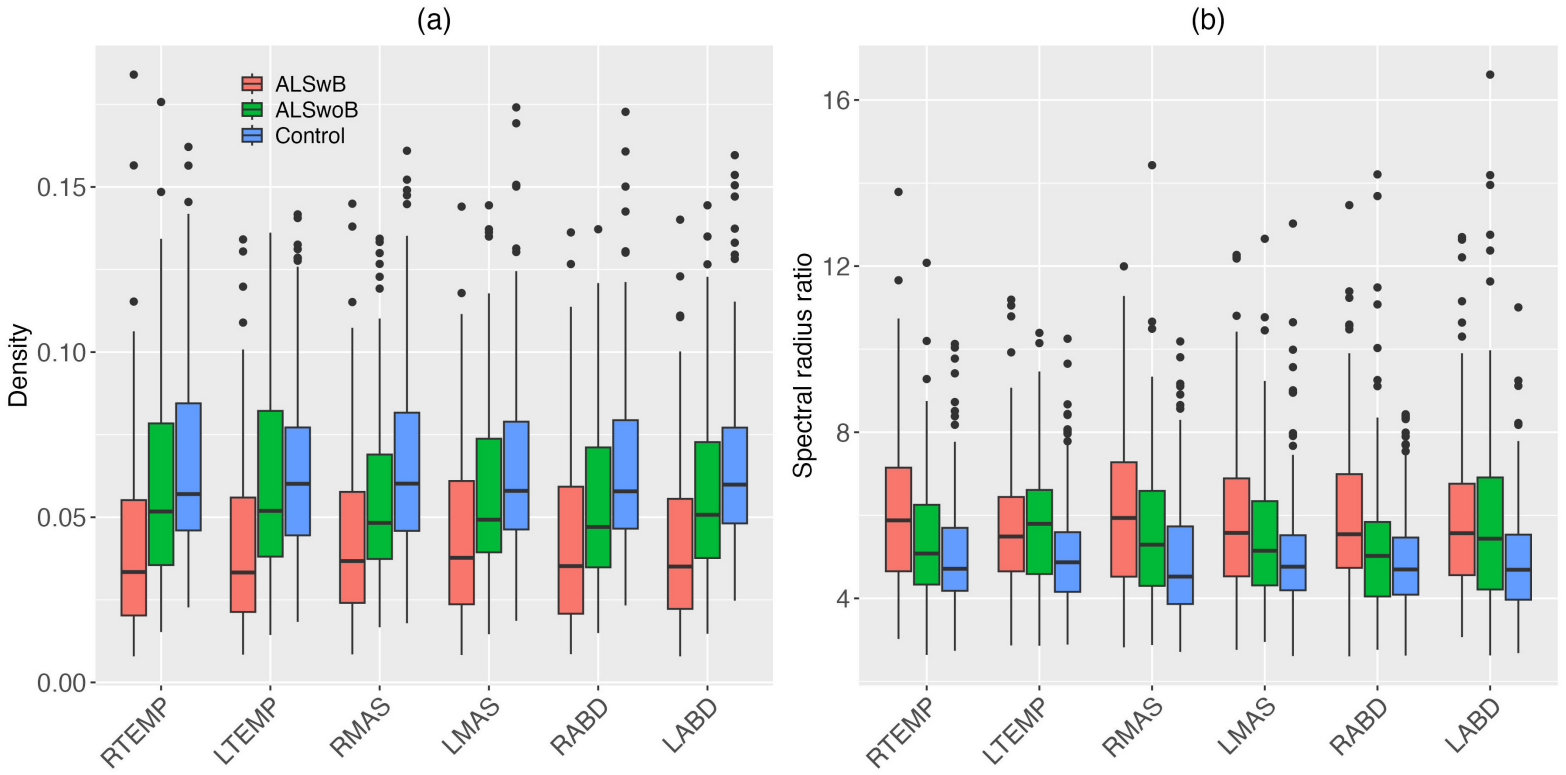
Boxplots for nodal features (density, spectral radius ratio) of the multiplex functional muscle network, displayed by node (RTEMP, right temporalis; LTEMP, left temporalis; RMAS, right masseter; LMAS, left masseter; RABD, right anterior belly of digastric; LABD, left anterior belly of digastric) and subgroup (ALSwB, individuals with amyotrophic lateral sclerosis, with overt clinical bulbar symptoms; ALSwoB, individuals with amyotrophic lateral sclerosis, absent of overt clinical bulbar symptoms; Control, healthy controls).

Descriptive boxplots and statistical results of the LME models for the nodal features are provided in Figure 4 and Table 3, respectively. Note that the descriptive boxplots are displayed for each subgroup of the ALS cohort (i.e., corresponding to the prodromal and symptomatic stages of bulbar involvement) alongside the healthy controls, to allow for visual examination of stage-dependent changes in these features. For both density and spectral radius ratio, a significant main effect was found for group, but not for node, nor did the interaction between group and node show a significant effect. *Post-hoc* comparisons between the ALS and healthy control groups revealed a significant decrease in density and a significant increase in spectral radius ratio, for all nodes. These results implied reduced level and increased variability of myoelectric activities in individuals with ALS relative to healthy controls. Most of these disease effects were visually identifiable as early as in the prodromal stage and were incremental as bulbar involvement progressed from prodromal to symptomatic stages, as shown in Figure 4.

**Table 3:** Statistics results of the linear mixed effects models for nodal features of the multiplex functional muscle network.

**Feature**	**Main effects and interaction**						***Post-hoc*** **comparison: ALS vs. control**											
	**Group**		**Node**		**Group** × **node**		**RTEMP**		**LTEMP**		**RMAS**		**LMAS**		**RABD**		**LABD**	
	***F***	***p***	***F***	***p***	***F***	***p***	***t***	***p***	***t***	***p***	***t***	***p***	***t***	***p***	***t***	***p***	***t***	***p***
density	7.78	**0.010**	0.21	0.96	0.12	0.99	−2.62	**0.014**	−2.50	**0.018**	−2.67	**0.012**	−2.46	**0.020**	−2.72	**0.011**	−2.79	**0.0091**
specradRatio	8.43	**0.0080**	0.61	0.69	1.16	0.33	2.44	**0.020**	2.33	**0.026**	3.02	**0.0048**	2.16	**0.038**	2.36	**0.024**	3.46	**0.0015**

ALS, amyotrophic lateral sclerosis; Control, healthy controls; RTEMP, right temporalis; LTEMP, left temporalis; RMAS, right masseter; LMAS, left masseter; RABD, right anterior belly of digastric; LABD, left anterior belly of digastric; F, F value; p, p value (note that the p values for the post-hoc tests are Bonferroni-adjusted); specradRatio, spectral radius ratio. Significant effects are highlighted in bold.

**Figure 5:**
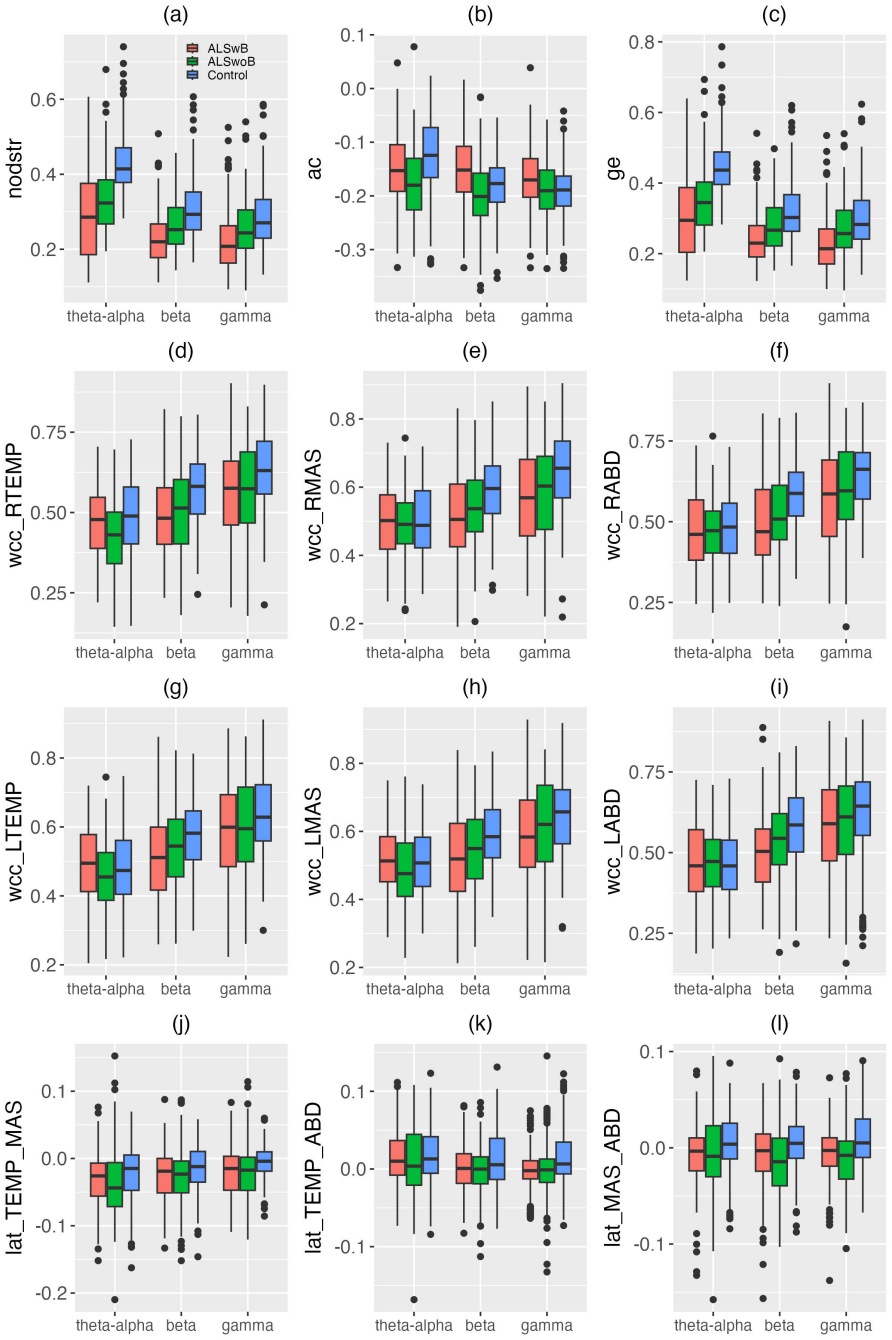
Boxplots for edgewise features (nodstr, ac, ge, wcc_RTEMP, wcc_RMAS, wcc_RABD, wcc_LTEMP, wcc_LMAS, wcc_LABD, lat_TEMP_MAS, lat_TEMP_ABD, lat_MAS_ABD) of the multiplex functional muscle network, displayed by band (theta/alpha, beta, gamma) and subgroup (ALSwB, individuals with amyotrophic lateral sclerosis, with overt clinical bulbar symptoms; ALSwoB, individuals with amyotrophic lateral sclerosis, absent of overt clinical bulbar symptoms; Control, healthy controls). nodstr, mean nodal strength; ac, assortativity coefficient; ge, global efficiency; wcc, weighted clustering coefficient; lat, laterality index. RTEMP, right temporalis; LTEMP, left temporalis; RMAS, right masseter; LMAS, left masseter; RABD, right anterior belly of digastric; LABD, left anterior belly of digastric.

Descriptive boxplots and statistical results of the LME models for the edgewise features are provided in Figure 5 and Table 4. Similar as above, the descriptive boxplots are displayed for each subgroup of the ALS cohort alongside the healthy controls. Based on the LME models, mean nodal strength, global efficiency, and the weighted clustering coefficients for right temporalis, right masseter, and right digastric, as well as the laterality index for the masseter-digastric group exhibited a significant main effect of group. All features except the laterality index for the masseter-digastric group showed a significant main effect of band. All features except the laterality index for the temporalis-masseter group and the laterality index for the masseter-digastric group revealed a significant effect of group-by-band interaction.

**Table 4:** Statistics results of the linear mixed effects models for edgewise features of the multiplex functional muscle network.

**Feature**	**Main effects and interaction**						***Post-hoc*** **comparison: ALS vs. control**					
	**Group**		**Band**		**Group** × **band**		**Theta/alpha**		**Beta**		**Gamma**	
	***F***	***p***	***F***	***p***	***F***	***p***	***t***	***p***	***t***	***p***	***t***	***p***
nodstr	13.53	**0.0012**	295.30	**< 0.001**	29.18	**< 0.001**	−5.46	**< 0.001**	−3.03	**0.0055**	−2.18	**0.039**
ac	0.22	0.64	74.58	**< 0.001**	34.57	**< 0.001**	−2.77	**0.0099**	0.54	0.59	−0.87	0.39
ge	13.64	**0.0012**	303.08	**< 0.001**	29.46	**< 0.001**	−5.55	**< 0.001**	−2.95	**0.0065**	−2.18	**0.038**
wcc_RTEMP	5.65	**0.026**	178.43	**< 0.001**	4.13	**0.016**	−1.35	0.19	−2.83	**0.0085**	−2.58	**0.015**
wcc_LTEMP	2.27	0.15	198.66	**< 0.001**	6.85	**0.0011**	−0.27	0.79	−2.35	**0.026**	−1.65	0.11
wcc_RMAS	4.93	**0.037**	136.94	**< 0.001**	11.67	**< 0.001**	−0.43	0.67	−2.85	**0.0078**	−2.97	**0.0059**
wcc_LMAS	2.76	0.11	151.05	**< 0.001**	5.10	**0.0062**	−0.59	0.56	−2.30	**0.029**	−1.83	0.078
wcc_RABD	4.38	**0.048**	197.41	**< 0.001**	15.98	**< 0.001**	−0.32	0.76	−3.17	**0.0037**	−2.49	**0.019**
wcc_LABD	0.71	0.41	184.99	**< 0.001**	8.41	**< 0.001**	0.061	0.95	−1.76	0.090	−0.74	0.46
lat_TEMP_MAS	2.32	0.14	21.55	**< 0.001**	1.06	0.35	−1.102	0.28	−1.41	0.17	−1.85	0.075
lat_TEMP_ABD	2.83	0.11	9.83	**< 0.001**	3.46	**0.032**	−0.73	0.47	−1.64	0.11	−2.35	**0.026**
lat_MAS_ABD	7.28	**0.013**	1.28	0.28	2.36	0.095	−1.66	0.11	−2.62	**0.013**	−3.14	**0.0035**

ALS, amyotrophic lateral sclerosis; Control, healthy controls; F, F value; p, p value (note that the p values for the post-hoc tests are Bonferroni-adjusted); nodstr, mean nodal strength; ac, assortativity coefficient; ge, global efficiency; wcc, weighted clustering coefficient; lat, laterality index. RTEMP, right temporalis; LTEMP, left temporalis; RMAS, right masseter; LMAS, left masseter; RABD, right anterior belly of digastric; LABD, left anterior belly of digastric. Significant effects are highlighted in bold.

*Post-hoc* comparisons between the ALS and healthy control groups showed a variety of significant cross-band and band-specific changes in the edgewise features, including (1) reduced mean nodal strength and global efficiency across all bands; (2) reduced theta/alpha-band assortativity coefficient; (3) reduced beta- and gamma-band weighted clustering coefficients for right temporalis, right masseter, and right digastric, as well as reduced beta-band weighted clustering coefficients for left temporalis and left masseter; (4) reduced beta- and gamma-band laterality for the masseter-digastric group and reduced gamma-band laterality for the temporalis-digastric group. As shown in Figure 5, all these disease effects were observed as early as in the prodromal stage; as bulbar involvement progressed from prodromal to symptomatic stages, mean nodal strength and global efficiency exhibited incremental changes upon the earlier-observed disease effects, whereas all other features revealed a trend of stabilization.

#### 3.2.2 Classification between ALS and healthy controls

**Figure 6:**
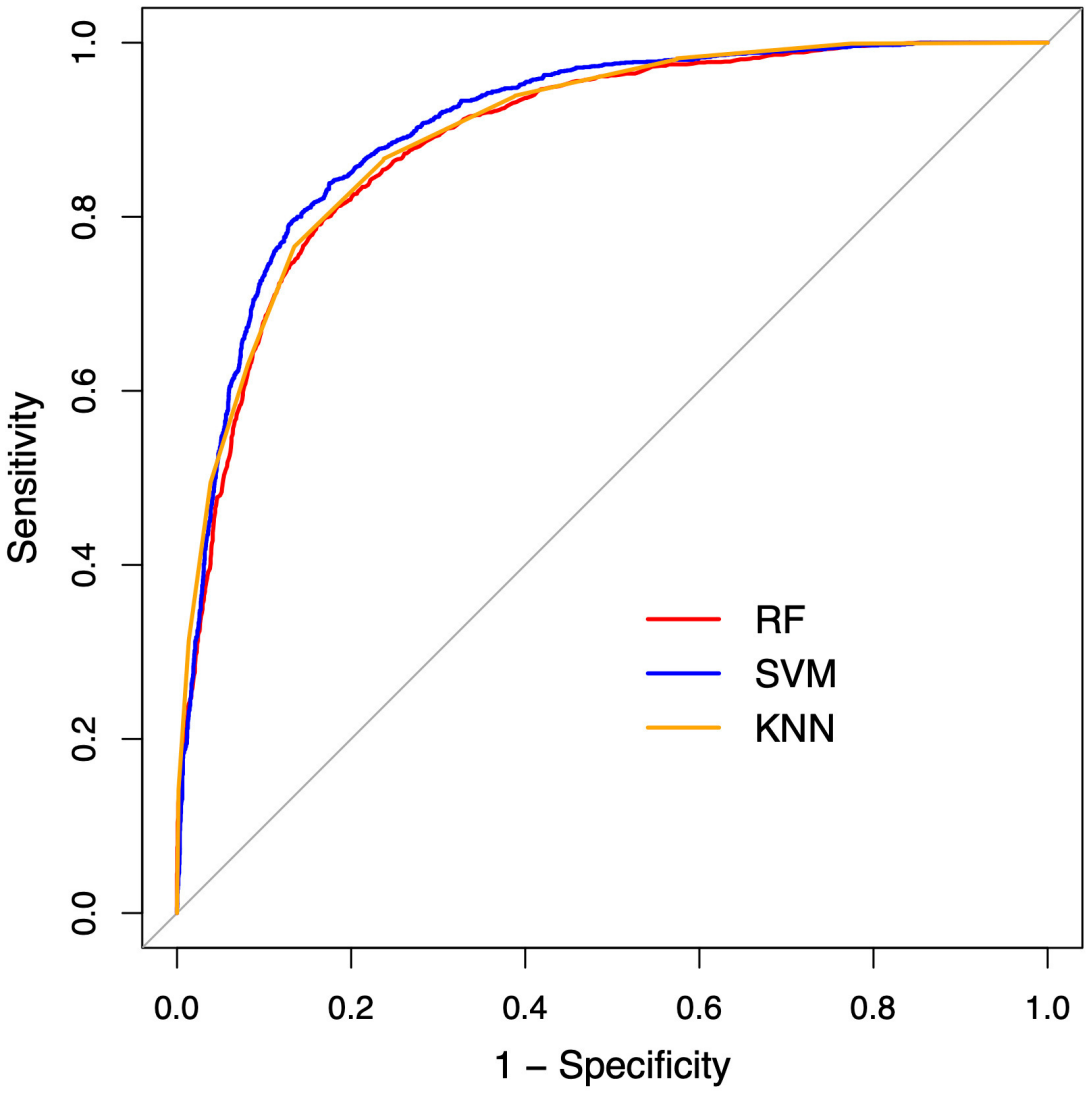
Receiver operating characteristic (ROC) curves for the machine learning classification models between amyotrophic lateral sclerosis and healthy control samples. RF, random forest; SVM, support vector machine; KNN, k-nearest neighbors.

The ROC curves and performance metrics of the classification models are provided in Figure 6 and Table 5, respectively. All three ML algorithms showed consistent performance, rendering an AUC of 0.89–0.91 and an F1 score of 0.85–0.87 with 0.84–0.86 precision and 0.87–0.88 recall for the classification between the ALS and healthy control samples. These results confirmed the robustness of the classification performance, providing evidence for the utility of the multiplex functional muscle network for detecting bulbar involvement in ALS across the prodromal and symptomatic stages with acceptably high efficacy.

**Table 5:** Classification performance of machine learning (ML) algorithms.

**ML algorithm**	**AUC**	**F1**	**Precision**	**Recall**
RF	0.89	0.85	0.84	0.87
SVM	0.91	0.87	0.86	0.88
KNN	0.90	0.86	0.85	0.87

AUC, area under the curve; RF, random forest; SVM, support vector machine; KNN, k-nearest neighbors.

### 3.3 Relation of the multiplex functional muscle network to behavioral patterns of the jaw

**Figure 7:**
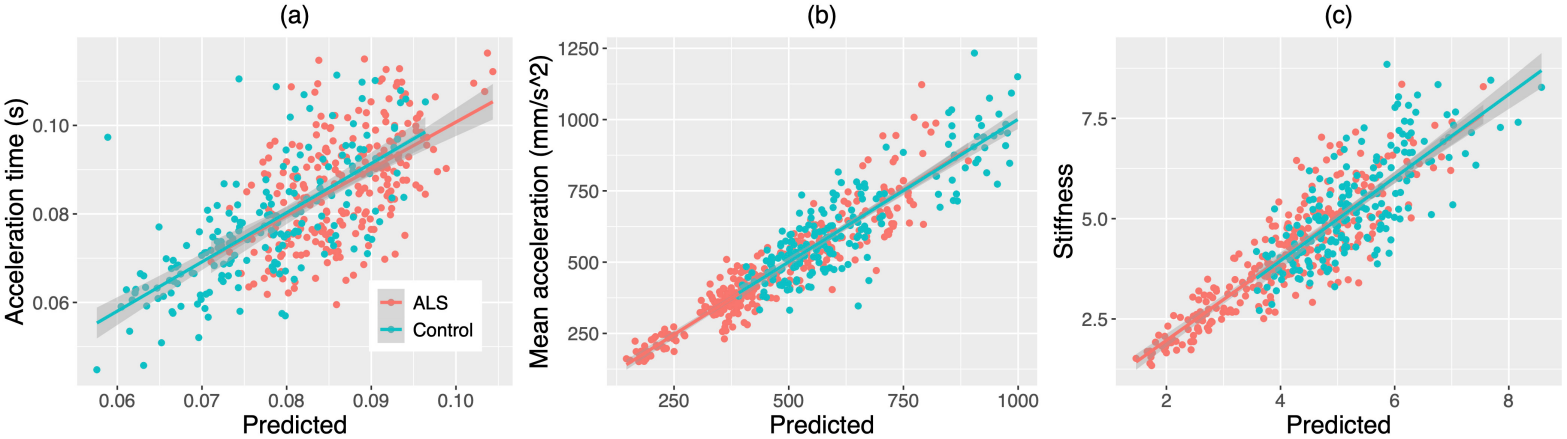
Scatterplots for **(A)** acceleration time, **(B)** mean acceleration, and **(C)** stiffness of the jaw against the predictions of the stepwise linear mixed effects models based on the factor scores of the multiplex functional muscle network. The lines and shaded regions around the lines are linear fits and 95% confidence intervals for individuals with amyotrophic lateral sclerosis (ALS, red) and healthy controls (Control, blue), respectively.

A subset of variables was identified by the stepwise LME models as predictors of each kinematic/dynamic metric of the jaw. The relationship between the model prediction and each metric is depicted in Figure 7. The composition and fit of the LME models are reported in the following.

Three latent factors of the functional muscle network were identified as predictive of acceleration time. These predictors included factor 1 (i.e., nodal features), *t* = −4.21, *p* < 0.001; factor 5 (i.e., theta/alpha-band mean nodal strength and theta/alpha-band global efficiency), *t* = 2.55, *p* = 0.011; and factor 9 (i.e., gamma-band mean nodal strength and gamma-band global efficiency), *t* = 2.13, *p* = 0.034. In addition, group was also selected as a predictor of the model (*p* = 0.020). The marginal and conditional *R*^2^ of the model were 0.16 and 0.45, respectively.

Four latent factors of the functional muscle network were identified as predictors of mean acceleration. These predictors included factor 1 (i.e., nodal features), *t* = 7.62, *p* < 0.001; factor 5 (i.e., theta/alpha-band mean nodal strength and theta/alpha-band global efficiency), *t* = −5.45, *p* < 0.001; factor 6 (i.e., theta/alpha-, beta-, and gamma-band laterality indices for the antagonist muscle groups), *t* = 3.42, *p* < 0.001; and factor 9 (i.e., gamma-band mean nodal strength and gamma-band global efficiency), *t* = −3.66, *p* < 0.001. The marginal and conditional *R*^2^ of the model were 0.073 and 0.81, respectively.

Five latent factors of the functional muscle network were identified as predictors of jaw stiffness. These predictors included factor 1 (i.e., nodal features), *t* = 14.30, *p* < 0.001; factor 5 (i.e., theta/alpha-band mean nodal strength and theta/alpha-band global efficiency), *t* = −5.31, *p* < 0.001; factor 6 (i.e., theta/alpha-, beta-, and gamma-band laterality indices for the antagonist muscle groups), *t* = 2.56, *p* = 0.011; factor 7 (i.e., beta-band mean nodal strength and beta-band global efficiency), *t* = −6.29, *p* < 0.001; and factor 9 (i.e., gamma-band mean nodal strength and gamma-band mean global efficiency), *t* = −10.53, *p* < 0.001. Moreover, group was selected as an additional predictor (*p* = 0.024). The marginal and conditional *R*^2^ of the model were 0.48 and 0.69, respectively.

## 4 Discussion

Developed upon a network-based neurodegeneration perspective, this study constructed a multiplex functional mandibular muscle network to link the clinical and neuropathological aspects of bulbar involvement in ALS, aiming to provide a novel objective tool for early identification and phenotyping of bulbar involvement. To this purpose, a clinically readily available, noninvasive electrophysiological technique—sEMG—was combined with graph network analysis to extract 48 features for assessing the performance of the muscle network. These features revealed a variety of disease-related changes, reflecting reduced and more variable myoelectric activation of all muscles, reduced functional connectivity and integration of the whole muscle network across three frequency bands (i.e., theta/alpha, beta, low gamma), and reduced functional specialization and lateralization of selective muscles in the beta and low gamma bands. Through dimension reduction, these features were successfully clustered into 10 interpretable latent factors, each reflecting a specific functional underpinning of the muscle network. These latent factors (1) demonstrated promise for detecting both clinically overt and silent bulbar neuromuscular changes across the prodromal and symptomatic stages and (2) were correlated with clinically meaningful behavioral changes of the jaw in individuals with ALS. These findings pave the way for applying the network-based neurodegeneration perspective and graph network analysis to evaluate bulbar involvement—a previously underexplored functional domain in neurodegenerative diseases that lacks a validated objective marker. Given the noninvasiveness and clinically readily availability of the instrument, and the fully automated data processing and analytics, the sEMG-based functional mandibular muscle network demonstrates strong potential for a clinically scalable efficient measurement tool to assess, monitor, and phenotype bulbar involvement in ALS.

### 4.1 Disease effects on the multiplex functional mandibular muscle network

The comparison of the network features between the ALS and healthy control groups reveals a variety of interpretable changes in line with the neuropathology of ALS, relating to both upper and lower motor neuron (UMN/LMN) degeneration. The combination of these features may therefore provide an effective means to profile bulbar involvement in ALS.

#### 4.1.1 Nodal features

Decreased density and increased spectral radius ratio are observed for all nodes/muscles in individuals with ALS (Table 3). As shown in Figure 4, these changes appear to occur as early as in the prodromal stage and decline incrementally from the prodromal to the symptomatic stages. Such descriptive stage-dependent changes along with the statistical results in Table 3 together reveal an early and incremental disease effect on the regulatory mechanisms of all muscles in the network, resulting in a trend toward progressively reduced and more variable myoelectric activities.

Reduced myoelectric activities can be attributed to various neurophysiological factors, including (1) decreased voluntary recruitment and firing rate of motor units (MUs) due to UMN involvement, (2) loss of viable MUs and altered muscle fiber conduction velocity due to denervation and compensatory reinnervation related to LMN involvement, and (3) reduced MU synchronization due to both UMN and LMN involvement (de Carvalho et al., 2014, 2012; Zwarts et al., 2000). Using a traditional linear amplitude analysis, Rong and Pattee (2022) has reported a similar decrease in the myoelectric activity of masseter but not of temporalis or anterior belly of digastric in individuals with ALS. The present finding of decreased myoelectric activities of all three muscles (on both sides) suggests that graph network analysis may provide a more sensitive means for detecting the effect of bulbar involvement on the modulation of myoelectric activities compared to linear analysis. Increased variability of myoelectric activities could be attributed to more variable firing rates, contractile properties, and conduction velocities of MUs due to LMN-related denervation and compensatory reinnervation (de Carvalho et al., 2014; Hansen and Ballantyne, 1978; Wohlfart, 1957). Such an increase in variability is in line with previous finding of decreased regularity of jaw muscle activities in individuals with ALS (Rong and Pattee, 2022).

#### 4.1.2 Edgewise features

Of all edgewise features, mean nodal strength and global efficiency appear to be the most robustly affected by ALS, exhibiting significant decreases across all frequency bands (i.e., theta/alpha, beta, low gamma). Furthermore, as shown by the descriptive boxplots in Figures 5A, C, these decreases are observed as early as in the prodromal stage and show incremental trends during the transition from the prodromal to the symptomatic stages. These results together elucidate an early and incremental disease effect on the overall functional connectivity and integration of the multiplex mandibular muscle network.

The disease effect on functional connectivity can be attributed to the impairment of multiple neural drives originating from motor cortical (i.e., beta and gamma oscillations) and other (i.e., theta/alpha oscillations) sources. Reduced beta functional connectivity has been previously reported for both bulbar and limb muscle groups in individual with ALS (Fisher et al., 2012; Issa et al., 2017; Rong and Pattee, 2021). Such changes have been suggested to reflect corticobulbar/corticospinal involvement, which impairs the motor cortical drive for synchronous oscillatory modulation of functionally related muscles during motor acts. The results of the current study resonate with and further extend these prior findings, suggesting that, in addition to beta oscillations, gamma and theta/alpha oscillations are also affected by ALS, leading to a global decrease in theta/alpha, beta, and gamma functional connectivity. Similar to beta functional connectivity, reduced gamma functional connectivity can also be interpreted as resulting from impaired motor cortical drive due to corticobulbar involvement, although the specific functional roles of beta and gamma oscillations likely differ in the time course of a motor act (Reed et al., 2021). Theta/alpha oscillations have been associated with sources outside of the motor cortex. A possible source is from proprioceptive afferents. Such afferents, as generated by the proprioceptors in muscles, are sent to the somatosensory cortex (S1) and then to the motor cortex (M1), together constituting a M1-muscle-S1-M1 feedback loop (Maezawa, 2017). The impairment of this proprioceptive feedback loop may underlie the decrease in theta/alpha functional connectivity in individuals with ALS.

In addition to the impairment of the central neural drives as noted above, physiological and histochemical changes in the muscles themselves (e.g., related to denervation and reinnervation) can also impact functional connectivity. Therefore, the observed decreases in functional connectivity are likely reflective of the interplay of all related central and peripheral factors, together leading to a global weakening of nodal linkage of the whole muscle network. The weakened linkage between muscles can further result in a functional disintegration of the muscle network, which is evidenced by the decreases in global efficiency across all three frequency bands in individuals with ALS.

The disease effects on the other edgewise features are less consistent across frequency bands. Weighted clustering coefficients only reveal significant decreases in the beta and gamma bands, but not in the theta/alpha band. Decreased weighted clustering coefficients imply less specialized functions of the muscles, which are likely attributed to the interplay of various pathological and compensatory factors. Pathologically, it is known that reduced voluntary recruitment (e.g., due to impaired beta and gamma cortical drives) and loss of viable MUs (e.g., due to LMN involvement) can differentially impair muscles with different physiological origins and compositions (DePaul et al., 1988; Rong and Jawdat, 2021; Rong and Pattee, 2021, 2022). In response to such differential impairments, individuals with ALS often make spontaneous adaptations to leverage the functional capacities of different muscles and use the less affected muscles to compensate for the functional impairments of the more affected ones. For instance, Rong and Pattee (2022) has reported an adaptive shift of primary agonist for speech-related jaw movement from masseter to temporalis in patients with ALS. This adaptation is intended to take advantage of the relative resistance of temporalis to neurodegeneration (due to its higher composition of slow fatigue-resistant fibers) (Frey et al., 2000; Pun et al., 2006), allowing patients to maintain the overall force generation capacity of the agonists despite the impairment of masseter. Yet, an adaptation like this inevitably reduces the functional specialization of different muscles (e.g., temporal vs. masseter), providing a possible explanation for the decreases in weighted clustering coefficients as observed in this study.

Similar as with the weighted clustering coefficients, the disease-related decreases in the laterality indices are also only observed in the beta and gamma bands, but not in the theta/alpha band. Such changes coincide with the prediction of the evolutionary neurodegeneration perspective discussed earlier, revealing a tendency toward preferential involvement of the motor cortex in the dominant left hemisphere. Given the known contralateral dominance of corticobulbar projection, such preferential left-sided cortical involvement would affect muscles on the right side more than those on the left, thereby diminishing the right-to-left laterality of muscle functioning as seen in Figures 5J–L. The finding that the laterality of the antagonist muscle groups is more affected than that of the agonist muscle group may be associated with the different functional roles of antagonists vs. agonists in speech production. While the coactivation of agonists generates large forces for jaw elevation, the coactivation of antagonists tunes the biomechanical properties of the mandibular system to facilitate the generation of rapid, precise jaw movement (Humphrey and Reed, 1983; Moore, 1993; Moore et al., 1988). Speech production typically does not demand large forces (compared to other oromotor tasks such as chewing) but requires rapid and precise articulatory movements to efficiently and accurately communicate linguistic messages. Such task demands may render antagonists a more important functional role than agonists in speech production, thereby enhancing their susceptibility to the disease effect.

### 4.2 Utility of the multiplex functional mandibular muscle network for detecting bulbar involvement in ALS

The factor analysis successfully clustered all 48 features of the multiplex functional mandibular muscle network into 10 interpretable latent factors, each representing a specific functional underpinning of the network. Based on the primary component features of each factor as identified in Figure 3, factors 1 to 10 can be interpreted as respectively reflecting (1) regulatory mechanisms of individual muscles; (2) gamma-band functional specialization; (3) theta/alpha-band functional specialization; (4) beta-band functional specialization; (5) theta/alpha-band functional connectivity and integration; (6) functional lateralization of the antagonist muscle groups; (7) beta-band functional connectivity and integration; (8) functional lateralization of the agonist muscle group; (9) gamma-band functional connectivity and integration; (10) assortativity/selective functional connectivity.

This factorization has two important implications: first, it reduces the dimensionality of the original features into a more manageable set of latent factors to circumvent the curse of dimensionality as commonly encountered in digital health applications (Berisha et al., 2021); second, the interpretability of these factors renders them suitable for objective markers of bulbar involvement. The second implication is supported by the results of the classification analysis. As shown in Table 5 and Figure 6, all classification models provide consistent evidence that the 10 latent factors combined can detect (subclinical) bulbar involvement across the prodromal and symptomatic stages with acceptably high efficacy. These findings demonstrate the promise of the multiplex functional mandibular muscle network for an objective tool to detect and measure both clinically overt and silent neuromuscular changes throughout the course of bulbar progression. Such a tool can effectively improve the early detection and monitoring of bulbar involvement in ALS.

### 4.3 Relation of the multiplex functional mandibular muscle network to clinically relevant behavioral patterns of the jaw

The latent factors of the multiplex functional mandibular muscle network were found to selectively correlate with the jaw kinematic/dynamic metrics during speech. As a key dynamic control variable, stiffness is known to be modulated by the neuromuscular system for regulating the rate and precision of movement (Humphrey and Reed, 1983; Moore, 1993; Moore et al., 1988). The findings of this study identify several neuromuscular factors for such stiffness modulation. These factors, including the level and variability of myoelectric activities (factor 1), the functional connectivity and integration of the whole multiplex muscle network (factors 5, 7, 9), and the functional lateralization of the antagonist muscle groups (factor 6), collectively account for 69% of the total variance in jaw stiffness. Notably, the majority of these factors (i.e., factors 1, 5, 9) also correlate with the acceleration time and mean acceleration of the jaw. Based on the directions of these correlations, the disease-related changes in the factors are associated with reduced stiffness, increased acceleration time, and reduced mean acceleration. Such changes are in alignment with the commonly reported behavioral deficits in ALS, including weakness, imprecision, and slowness (Bandini et al., 2018; Darley et al., 1969a,b; Moore and Rong, 2022; Rong and Heidrick, 2021; Rong et al., 2021, 2015a, 2016; Shellikeri et al., 2016; Yunusova et al., 2010).

The mutual contributions of the above-identified neuromuscular factors to all three dynamic/kinematic metrics elucidate a possible explanatory model of speech production. In this model, stiffness, as an implicit dynamic control variable, serves as a mediator to connect the muscle network to the kinematic properties of the effector (i.e., acceleration time and mean acceleration). This model provides novel and much-needed insights into the clinical-neuropathological relationship, by linking between the neuromuscular underpinnings (muscle network), the dynamic control mechanism (stiffness), and the behavioral manifestations (acceleration profile) of bulbar involvement in ALS. Such a model warrants further investigation in future work. Lastly, it is worth noting that all predicted relationships between the neuromuscular factors and kinematic/dynamic metrics are followed by the data for both individuals with ALS and healthy controls, as shown in Figure 7. This observation provides supportive evidence to rule out a common “third variable”—disease severity—which underlies and often confounds the relationship between the predictors and the outcome in correlation analysis on data drawn from a population with a wide range of severity (Weismer, 2006).

### 4.4 Clinical implications

Using fully automated data processing and analytic procedures, this study constructed a sEMG-based multiplex functional mandibular muscle network for objective assessment and profiling of bulbar involvement in ALS. This network approach has various strengths over the existing clinical and quantitative tools for bulbar assessment. Compared with the standard clinical tools which rely primarily on expert-guided subjective evaluation of overt clinical symptoms and functional declines, our network approach detects and quantifies not only overt bulbar involvement during the symptomatic stage, but also clinically silent bulbar neuromuscular changes during the prodromal stage. Neurodegenerative diseases are known to have a long prodromal stage, during which a variety of subclinical changes occur silently at different levels (Eisen et al., 2014). After years to decades of progression, these changes eventually culminate in clinical symptoms and functional declines. With the demonstrated sensitivity to prodromal bulbar neuromuscular changes, our network approach can effectively improve the early detection of bulbar involvement in ALS, providing a supplementary assessment tool to the existing clinical standards.

In addition to the standard clinical tools, a variety of instrumental approaches using contemporary kinematic (e.g., sensor-based or markerless facial motion tracking) and acoustic (e.g., lab-grade or mobile app-based speech recording) techniques exist, allowing for alternative means of quantitative bulbar assessment. These techniques, however, either rely on instruments that are currently unavailable in a clinical setting (e.g., kinematic tracking device) or focus on behavioral measures (e.g., acoustic features) that lack mechanistic insights into the neuropathological underpinnings. Compared with these techniques, our network approach utilizes a clinically readily available noninvasive instrument (i.e., sEMG) and generates a set of interpretable objective outcome measures (i.e., latent factors) that are both (1) explanatorily linkable to the neuromuscular mechanisms of bulbar involvement and (2) correlated with clinically meaningful behavioral changes (e.g., reduced stiffness, increased acceleration time, reduced mean acceleration). Moreover, the fully automated data processing and analytic procedures require minimal training and technical expertise, largely enhancing the scalability of the network approach to routine clinical practice.

With the various strengths as noted above, the network approach has a variety of clinical implications. First, the identification and quantification of clinically indiscernible subclinical bulbar neuromuscular changes during the prodromal stage would allow clinicians more time to engage patients and their caregivers into the conversation about the expected functional declines of the patients and the management options available to support informed decision-making and patient-centered care. Second, although not statistically tested, the descriptive patterns in Figures 4, 5 reveal a trend toward progressive involvement of various nodal (e.g., density, spectral radius ratio) and edgewise (e.g., mean nodal strength, global efficiency) features of the muscle network across the prodromal and symptomatic stages. With further statistical verification warranted, the latent factors associated with these features (e.g., factors 1, 5, 7, 9) may provide useful objective markers for monitoring bulbar disease progression to guide stage-dependent, tailored intervention for optimized outcomes. Third, the finding that the multiplex functional muscle network exhibits interpretable disease-related changes, which are linkable to both the neuromuscular mechanisms and behavioral patterns of bulbar involvement, corroborates the potential of the network for an objective tool to measure and phenotype bulbar involvement. Such phenotype information could improve (1) personalized care of bulbar dysfunction and (2) patient stratification for clinical trials to facilitate the efforts for therapeutic treatment discovery and early trial enrollment.

### 4.5 Limitations and future directions

As an initial effort to apply graph network analysis for assessing and characterizing bulbar involvement, there are several limitations of this study that should be leveraged in future work along similar lines. During network construction, target muscles were selected mainly based on accessibility consideration. While anterior temporalis, masseter, and anterior belly of digastric muscles are well supported by the evidence from the literature to be accessible and reliably measurable by surface electrodes, it should be noted that these muscles only consist of a small portion of the complex bulbar musculature. Thus, the generalizability of the findings to other muscles with distinct physiological origins and compositions remains unclear. Nevertheless, the methodology of this study, including the experimental paradigm and all data processing and analytic procedures, is adaptable for other bulbar muscles, enabling future scaling to larger-scale muscle networks.

In addition to the disease effects, various other factors that are unrelated or not directly relatable to the disease can influence the performance of the muscle network. These factors, including aging, fatigue, and technical specifications of the sEMG system, might confound the disease effects on the network properties such as functional connectivity. Such contextual factors should be taken into account in the study design and be better delineated and controlled in the data analysis in future larger-scale studies.

While we demonstrated that the disease-related changes in the network features can be explanatorily linked to the combined effects of UMN and LMN degeneration (along with other related neurodegenerative processes), these intertwined neuropathological factors remain hard to disentangle based on the current approach. This limitation, however, does not diminish the impact of developing a novel objective tool to comprehensively assess the complete picture of bulbar neuromuscular pathology in ALS, relating to both UMN and LMN degeneration. Such a tool is highly needed for the understanding, phenotyping, and tailored management of bulbar involvement.

Although our sample was carefully controlled in clinical characteristics such as disease onset (i.e., bulbar onset: 33%, resembling the population-wise prevalence of 25%−30%) and staging of bulbar involvement (i.e., roughly balanced between the prodromal [47%] and symptomatic [53%] cases), the limitations of the sample size and population diversity due to the single-site recruitment raise caution to generalizing the findings to larger patient populations. Additionally, given the small number of samples after stratifying the ALS patients into the prodromal and symptomatic subgroups, we did not conduct statistical analysis on the stratified samples. Thus, the stage-dependent changes in all network features, as illustrated in Figures 4, 5, are descriptive and require further statistical verification. To address these limitations, a cross-site external validation with larger, independent, and more heterogenous ALS cohorts is needed to substantiate the clinical utility of the proposed network approach in diverse patient populations and subgroups.

The dataset analyzed in this study is cross-sectional in nature. The discussion on the effect of bulbar progression on the muscle network is based on the comparison between individuals at different stages of bulbar involvement rather than the dynamic changes within an individual throughout their disease course; the relevant implication for progress monitoring is thus speculative. The heterogeneity in bulbar progression trajectories is well recognized in ALS (Rong et al., 2020, 2015b). To characterize and potentially stratify such progression trajectories, a well-powered longitudinal dataset is needed in future research to (1) evaluate the responsiveness of the muscle network to bulbar progression within an individual and (2) identify potential subtypes (e.g., fast vs. slow progressors) to facilitate disease prognosis and intervention planning.

Although we identified significant correlations of the latent factors of the muscle network to acceleration time and mean acceleration, it should be noted that the marginal *R*^2^ of both models (0.073 − 0.16) are substantially lower than the conditional *R*^2^ (0.45 − 0.81). This observation suggests that a notable proportion of variance in these kinematic metrics cannot be explained by the fixed effects of the models. Additional latent variables likely exist alongside the factors examined in this study to jointly contribute to the acceleration profile of the jaw. For example, other muscles (e.g., medial pterygoid) may play a role in regulating jaw kinematics in addition to temporalis, masseter, and digastric. Such unknown variables remain to be identified in future research.

In a prior study, Rong and Pattee (2022) has applied traditional linear analysis in both frequency and time domains along with recurrence quantification analysis to assess the amplitude, frequency, complexity, regularity, and coordination of jaw muscle activities. Various features have been identified as being sensitive to early bulbar involvement in ALS. These features provide distinctive insights into the neuromuscular pathology of bulbar involvement that are complementary to the network features in this study. One future direction is to explore the possibility of combining the analytic approaches in the current and previous studies into a comprehensive multi-construct assessment protocol to further improve the detection and phenotyping of bulbar involvement in ALS.

## 5 Conclusions

To our knowledge, this was the first study to adopt the concept of network-based neurodegeneration, which has been primarily applied to brain networks associated with dementia syndromes in previous studies, to construct a multiplex functional mandibular muscle network for assessing bulbar involvement—a hallmark feature of ALS currently lacking objective markers. This network was constructed based on a clinically readily available noninvasive electrophysiological technique—sEMG—coupled with fully automated data processing and analytic procedures to extract 48 features reflecting the network properties during a speech task. These features exhibited a variety of changes in individuals with ALS, all being explanatorily linkable to the neuropathology of ALS, related to both UMN and LMN degeneration. Through dimension reduction, the features were successfully clustered into 10 interpretable latent factors, each reflecting a specific functional underpinning of the muscle network.

To evaluate the utility of the latent factors of the muscle network as objective markers of bulbar involvement, statistical and ML algorithms were trained and tested on a heterogenous ALS dataset, consisting of about half samples presenting with overt clinical bulbar symptoms and half without, along with a matched healthy control sample set. Based on these analyses, the latent factors (1) demonstrated efficacy for detecting both clinically overt and silent neuromuscular changes across the prodromal and symptomatic stages of bulbar involvement, and (2) were correlated with clinically meaningful behavioral changes in the jaw (i.e., reduced stiffness, increased acceleration time, reduced mean acceleration). These findings provide converging supportive evidence for the multiplex functional mandibular muscle network to serve as an objective measurement tool to improve the early detection and profiling of bulbar involvement in ALS. This tool has several notable strengths, including the use of a clinically readily available noninvasive instrument, fully automated data processing and analytics, and the generation of clinically relevant, interpretable objective outcome measures. These strengths render the tool highly scalable for a variety of clinical applications to improve personalized management and clinical trial planning for individuals with ALS.

## Data Availability

The raw data supporting the conclusions of this article will be made available by the authors, without undue reservation.
