# Supplementary material for: A novel muscle network approach for objective assessment and profiling of bulbar involvement in ALS
Source: Front Neurosci. 2025 Jan 10;18:1491997. doi: 10.3389/fnins.2024.1491997 (PMC11759300; doi:10.3389/fnins.2024.1491997)
Supplement: Supplementary file 1 [file Table_1.docx]

Table S1. Demographic, clinical, and functional characteristics of participants. Descriptive statistics are provided as mean (standard deviation) by group at the bottom of the table. Gender: M = male, F = female. Onset: B = bulbar, C = cervical, L = lumbar, N = neck. DaysSinceDiag = disease duration in days since diagnosis. Tot_ALSFRS = total score on the Amyotrophic Lateral Sclerosis Functional Rating Scale-Revised (maximum score = 48). Bulb_ALSFRS = bulbar subscore on the Amyotrophic Lateral Sclerosis Functional Rating Scale-Revised (maximum score = 12). Group: Control = healthy controls; ALSwoB = individuals at the prodromal stage of bulbar involvement secondary to amyotrophic lateral sclerosis, absent of overt clinical bulbar symptoms; ALSwB = individuals at the symptomatic stage of bulbar involvement secondary to amyotrophic lateral sclerosis, presented with overt clinical bulbar symptoms. All participants were nonsmokers, who either had no smoking history or quit smoking for more than one year.

| **SubjectID** | **Gender** | **Age** | **Onset** | **DaysSinceDiag** | **Tot_ALSFRS** | **Bulb_ALSFRS** | **Group** |
| --- | --- | --- | --- | --- | --- | --- | --- |
| ALS1 | M | 73 | B | 134 | 42 | 10 | ALSwB |
| ALS2 | M | 47 | C | 1668 | 43 | 12 | ALSwoB |
| ALS3 | M | 74 | C/N | 87 | 37 | 11 | ALSwoB |
| ALS4 | M | 72 | L | 407 | 45 | 12 | ALSwoB |
| ALS5 | F | 65 | B | 266 | 39 | 10 | ALSwB |
| ALS6 | M | 58 | L | 56 | 42 | 12 | ALSwoB |
| ALS7 | F | 66 | C | 294 | 24 | 11 | ALSwB |
| ALS8 | F | 62 | B | 150 | 24 | 4 | ALSwB |
| ALS9 | M | 38 | L | 123 | 40 | 11 | ALSwB |
| ALS10 | M | 38 | C/L | 491 | 33 | 11 | ALSwoB |
| ALS11 | M | 52 | C | 192 | 42 | 10 | ALSwoB |
| ALS12 | F | 73 | B | 571 | 32 | 6 | ALSwB |
| ALS13 | F | 56 | C | 269 | 37 | 11 | ALSwoB |
| ALS14 | M | 60 | L | 869 | 43 | 10 | ALSwB |
| ALS15 | F | 77 | B | 167 | 37 | 3 | ALSwB |
| HC1 | F | 55 | n/a | n/a | n/a | n/a | Control |
| HC2 | F | 38 | n/a | n/a | n/a | n/a | Control |
| HC3 | M | 65 | n/a | n/a | n/a | n/a | Control |
| HC4 | M | 76 | n/a | n/a | n/a | n/a | Control |
| HC5 | M | 81 | n/a | n/a | n/a | n/a | Control |
| HC6 | F | 71 | n/a | n/a | n/a | n/a | Control |
| HC7 | F | 80 | n/a | n/a | n/a | n/a | Control |
| HC8 | F | 62 | n/a | n/a | n/a | n/a | Control |
| HC9 | F | 74 | n/a | n/a | n/a | n/a | Control |
| HC10 | F | 66 | n/a | n/a | n/a | n/a | Control |
| Control |  | 66.80 (13.02) |  | - | - | - |  |
| ALSwoB |  | 56.71 (12.92) |  | 452.86 (558.77) | 39.86 (4.26) | 11.29 (0.76) |  |
| ALSwB |  | 64.25 (12.14) |  | 321.75 (265.57) | 35.13 (7.64) | 8.13 (3.27) |  |
